# The Whole Genome DNA Methylation Signatures of Hindlimb Muscles in Chinese Alligators during Hibernation and Active Periods

**DOI:** 10.3390/ani14131972

**Published:** 2024-07-03

**Authors:** Jihui Zhang, Xiaobing Wu

**Affiliations:** 1 School of Food Science and Biology Engineering, Wuhu Institute of Technology, Wuhu 241000, China; jihui871031@163.com; 2 College of Life Sciences, Anhui Normal University, Wuhu 241000, China

**Keywords:** adaptation, *Alligator sinensis*, hibernation, DNA methylation

## Abstract

**Simple Summary:**

Hibernation is a physiological state for Chinese alligators to cope with cold weather. DNA methylation, an epigenetic factor discovered early and extensively studied, plays a crucial role in regulating gene expression, development, and stress responses. The role of DNA methylation in regulating gene expression related to hibernation in ectotherms remains unclear. Here, genome-wide DNA methylation maps of the hindlimb muscles of Chinese alligators were generated, and differential methylation genes and related metabolic pathways of the hindlimb muscles of Chinese alligators during hibernation and active periods were screened. These differential genes and metabolic pathways are closely related to lipid metabolism, energy metabolism, and amino acid metabolism. The findings suggest that DNA methylation may play a significant role in regulating the adaptive hibernation mechanisms of Chinese alligators. This study is helpful to further understand the molecular basis of hibernation adaptation in Chinese alligators.

**Abstract:**

Many ectotherms hibernate to increase their chances of survival during harsh winter conditions. The role of DNA methylation in regulating gene expression related to hibernation in ectotherms remains unclear. Here, we employed whole-genome bisulfite sequencing (WGBS) technology to construct a comprehensive genome-wide DNA methylation landscape of the hindlimb muscles in the Chinese alligator during hibernation and active periods. The results indicated that methylation modifications were most abundant at CG sites, identifying 9447 differentially methylated regions (DMRs) and 2329 differentially methylated genes (DMGs). KEGG pathway enrichment analysis of the DMGs revealed significant enrichment in major pathways such as the neurotrophin signaling pathway, the MAPK signaling pathway, the GnRH signaling pathway, the biosynthesis of amino acids, and the regulation of the actin cytoskeleton, which are closely related to lipid metabolism, energy metabolism, and amino acid metabolism. Among these, 412 differentially methylated genes were located in promoter regions, including genes related to energy metabolism such as ATP5F1C, ATP5MD, PDK3, ANGPTL1, and ANGPTL2, and genes related to ubiquitin-proteasome degradation such as FBXO28, FBXO43, KLHL40, and PSMD5. These findings suggest that methylation in promoter regions may play a significant role in regulating the adaptive hibernation mechanisms in the Chinese alligator. This study contributes to a further understanding of the epigenetic mechanisms behind the hibernation of the Chinese alligator.

## 1. Introduction

Chinese alligators (*Alligator sinensis*), a species endemic to China, were once on the brink of extinction. They predominantly inhabit regions between 30.6° and 31.6° north latitude and 118° and 119.6° east longitude. As poikilotherms, Chinese alligators experience a decrease in core body temperature when ambient temperatures fall below 10 °C. Consequently, they cease feeding and enter a hibernation state, with oxygen consumption for respiration reduced to 20–25% of normal activity levels [1]. Prior researchers have demonstrated that the quality of hibernation directly impacts the hibernation survival rate and subsequent year’s reproductive rate of Chinese alligators [2,3]. Our team conducted a detailed comparative study on the blood of Chinese alligators during hibernation and the active phase and found that the red blood cells, white blood cells, cholesterol, and triglycerides in the blood during hibernation were significantly reduced, and the mean corpuscular volume and the mean corpuscular hemoglobin were significantly increased [4]. So far, the seasonal adaptation of the Chinese alligator and its intestinal flora have been previously studied by transcriptome and metagenomics analysis [1,2,5]. Building on this foundation, we hope to further investigate the regulatory role of methylation on differentially expressed genes in the muscles of Chinese alligators during their hibernation and active phases. Today, in addition to human hunting and habitat disruption, global climate change is becoming a critical threat to Chinese alligators, as indeed it is for many other hibernators [6]. Therefore, investigating the gene regulatory network underlying seasonal physiological changes is of paramount importance not only in elucidating how hibernating ectotherms adapt to harsh winter conditions and limited food resources in their natural habitats but also potentially contributing to their future conservation efforts.

DNA methylation, first discovered in 1975 [7,8], remains one of the most extensively investigated and well-studied epigenetic factors. DNA methylation is an ancient epigenetic modification in eukaryotic genomes that plays essential roles in various biological processes, including the regulation of gene expression, development, and stress responses [9,10,11,12,13]. Previous research evaluated global changes in DNA methylation in response to hibernation in the liver and skeletal muscle of thirteen-lined ground squirrels, along with changes in the expression of DNA methyltransferases (DNMT1/3B) and methyl binding domain proteins (MBDs) [14]. In the brown adipose tissue of hibernating thirteen-lined ground squirrels, global transcriptional suppression is achieved through epigenetic regulatory mechanisms [15]. However, previous studies largely focused on changes in overall DNA methylation levels or the methylation of specific genes. Epigenetic factors, including miRNA and DNA methylation, play crucial roles in regulating gene expression and serve as pivotal regulators of skeletal muscle development, energy metabolism, and various other biological processes [16,17,18,19]. Previously, we investigated the regulatory mechanism of miRNA in the hindlimb muscles of adult Chinese alligators with regards to their adaptability during hibernation [20]. In this study, we employed whole-genome bisulfite sequencing (WGBS) to generate single-base resolution DNA methylation maps of the hindlimb muscles in hibernating and active Chinese alligators, aiming to elucidate the methylation changes in the hindlimb muscles of Chinese alligators during hibernation and activity phases.

The vertebrate skeletal muscle is a substantial tissue in the body, primarily responsible for bodily movement, energy metabolism, protein storage, safeguarding internal organs, and fulfilling other vital functions [21,22]. The weight of the hindlimb muscles of quadrupedal tetrapod species accounts for a large proportion of body weight. Compared with the hindlimbs of other vertebrate groups, the hindlimbs of alligators have stronger compliant muscles, which enhances their mobility [23,24,25]. Alligators are unusual among tetrapods in their ability to use a wide range of hindlimb postures, even over a restricted range of speeds [26,27]. Research conducted on the skeletal muscles of hibernating animals demonstrates that their skeletal muscles exhibit well-maintained strength and anti-fatigue capabilities throughout the period of hibernation [28,29,30]. A similar phenomenon is observed in the skeletal muscles of hibernating crocodiles, which enables them to rapidly resume active behaviors such as predator avoidance, foraging, and mating when they awaken in the spring [31]. In addition, the muscles of amphibians also show adaptability to hibernation. For example, their skeletal muscles have been shown to degrade to produce glycogen to meet their hibernation needs [32]. Hibernation is characterized by prolonged periods of inactivity with concomitantly low nutrient intake, conditions that would typically result in muscle atrophy combined with a loss of oxidative fibers. Yet, hibernators consistently emerge from winter with very little atrophy. The investigation of the unique adaptability of skeletal muscles during reptile hibernation holds significant value for academic research, thus warranting further exploration. Although skeletal muscle adaptations have been extensively investigated in hibernating species such as *Urocitellus richardsonii* [28], *Ursus arctos* [33], and *Spermophilus dauricus* [34], the study of hindlimb muscle adaptations during hibernation in reptiles remains limited to a few species [2,35,36]. The findings from this study will provide a fundamental foundation for gaining further insights into the adaptive regulatory mechanisms of hindlimb muscles in Chinese alligators during hibernation.

## 2. Materials and Methods

### 2.1. Sample Collection

As the Chinese alligator is a rare protected species endemic to China, the availability of specimens for scientific research is extremely limited. We obtained four adult female Chinese alligators from the Chinese Alligator Nature Reserve in Xuancheng City, Anhui Province, China. These adult Chinese alligators exhibited similar weight and length characteristics (Supplementary Table S1A). Two female specimens were collected in January 2018 during deep hibernation, with an average body temperature of approximately 8.9 °C. The other two female specimens were collected in June 2018 during the active phase, with an average body temperature of ca. 23.7 °C. Euthanasia of the four adult Chinese alligators was conducted via intravenous injection of pentobarbital (350 mg/kg). Muscle tissue from the hindlimb (the quadriceps of the hindlimb) was dissected for analysis. The collected samples (Supplementary Table S1B) were promptly frozen in liquid nitrogen and stored at −80 °C until further use. All animal experiments were conducted with approval from the animal ethics committee of Anhui Normal University, under a license number of 2018012.

### 2.2. DNA Extraction, Library Construction, and Sequencing

Genomic DNA was extracted from hindlimb muscle tissue samples of Chinese alligators during both hibernation and active periods using a genomic DNA extraction kit (TIANamp, Beijing, China). Subsequently, the purity and concentration of the DNA samples were measured using 1% agarose gel electrophoresis and the Qubit 2.0 fluorometer (Life Technologies, Carlsbad, CA, USA). We utilized 1 µg of high-quality genomic DNA and supplemented it with 1 ng of unmethylated lambda DNA as a negative control. The genomic DNA underwent random shearing to yield fragments ranging from 200 to 400 bp using Covaris M220, followed by repair and adenylation of the fragmented DNA ends. Adapters containing methylated cytosines were then ligated to the A-tailed DNA fragments. Subsequently, the DNA fragments, extracted from gel according to the EZ DNA Methylation Gold Kit (Zymo Research, Irvine, CA, USA) instructions, underwent bisulfite treatment. Following treatment, methylated cytosines on the genomic DNA remained unaltered, while unmethylated cytosines were converted to uracils (subsequently transformed to thymines after PCR amplification). Finally, the DNA library was enriched through PCR amplification. The amplified library was purified by 0.85× AMPure XP. After purification, Qubit 2.0 was used for preliminary quantification, and the library was diluted to 1 ng/µL. Then the fragment range and effective concentration of the library were measured by Agilent 2100 (Agilent, Waldbroon, Germany) and qPCR to ensure the quality of the library, which can be used for subsequent experiments. After the paired-end library construction, the libraries were sequenced on an Illumina HiSeq 2500 platform (Illumina, San Diego, CA, USA).

### 2.3. Sequencing Data Analysis

The raw sequencing data were filtered using the fastqc software (v0.11.9, https://www.bioinformatics.babraham.ac.uk/projects/fastqc/, accessed on 20 January 2021) to remove low-quality junk data, retain clean data, and ensure its quality. We trimmed the sequencing adapters and low-quality data from the raw sequencing data to obtain clean data for subsequent analysis [37]. Then, the clean reads were aligned to the Chinese alligator genome [38] using Bismap software (V2.90) with the default parameters. Conversions of C to T and G to A were separately made in the reference genome and sequence reads before alignment, and the bisulfite-converted genome was indexed by Bowtie2 (v2.4.0). The bisulfite non-conversion rate is determined by the proportion of cytosines sequenced at the cytosine reference site. With coverage of ≥ 5× and a false discovery rate of < 0.05, we performed a binomial distribution test for each C site to detect methylated cytosines [39,40].

### 2.4. Identification of DMRs and DMGs

We employed the Metilene software (v0.2.8) for analyzing differentially methylated regions (DMRs). This software employs a binary segmentation algorithm in conjunction with dual statistical tests (MWU-test and 2D KS-test) to effectively detect DMRs between paired samples or two groups of samples. Subsequently, DMRs are refined through multiple testing corrections. In this study, CpG sites were utilized to identify differentially methylated regions. The criteria for selecting differentially methylated regions were as follows: an average methylation level difference > 0.1, a false discovery rate (FDR) < 0.05 for differential testing between the two regions, and the presence of at least five methylated CpG sites within each DMR, with the distance between CpG sites being less than 300 bp. Based on the DMR annotation results, genes overlapping with DMRs in the genebody and its upstream and downstream regions (upstream 2 K, genebody, and downstream 2 K) were screened and defined as differentially methylated genes (DMGs). We defined the upstream 2000 bp of genes as the promoter region and used the same screening method to identify differentially promoter-methylated genes (DPMGs).

### 2.5. Gene Ontology and KEGG Pathway Analysis of DMGs and DPMGs

The DMGs and DPMGs were screened and annotated by gene ontology (GO) and Kyoto Encyclopedia of Genes and Genomes (KEGG) enrichment analyses. GO enrichment analysis was achieved using the GOseq R software (v3.19) package [41], in which GO items with a *p*-value < 0.05 were considered significantly enriched. KEGG can be used to analyze advanced functions and biological systems (such as cells, organisms, and ecosystems) at a molecular level (http://www.genome.jp/kegg/, accessed on 20 January 2021) [42]. KOBAS software (v3.0) was used to analyze the statistical enrichment of DMGs and DPMGs in KEGG pathways [43].

### 2.6. Bisulfite Sequencing PCR

Gene-specific DNA methylation was assessed by a next-generation sequencing-based BSP, according to a previously published method [44,45]. In brief, BSP primers were designed using the online MethPrimer software (v2.0) and listed in Supplementary Table S11. A total of 1 μg of genomic DNA was converted using the ZYMO EZ DNA Methylation-Gold Kit (Zymo Research, Irvine, CA, USA), and one twentieth of the elution products were used as templates for PCR amplification with 35 cycles using the KAPA 2G Robust HotStart PCR Kit (Kapa Biosystems, Wilmington, MA, USA). For each sample, BSP products of multiple genes were pooled equally, 5′-phosphorylated, 3′-dA-tailed, and ligated to the barcoded adapter using T4 DNA ligase (NEB). Barcoded libraries from all samples were sequenced on the Illumina platform.

For the bisulfite sequencing reads of each sample, adapters and low-quality reads were removed using the software Trimmomatic-0.36. After removing the adapter sequences and filtering out the low-quality reads, the clean sequencing reads were directly aligned to the target sequences using the software Bsmap (v2.73) with the default parameters, which combine genome hashing and bitwise masking to achieve fast and accurate bisulfite mapping. Methylation levels are defined as the fraction of read counts of ‘C’ in the total read counts of both ‘C’ and ‘T’ for each covered C site. On the basis of such a read fraction, methylated cytosine was shown using a binomial distribution as in the method described by Lister et al. [46], whereby a probability mass function is calculated for each methylation context (CpG, CHG, CHH). A two-tailed Fisher’s exact test was used to identify cytosines that are differentially methylated between two samples or groups. Only those CGs covered by at least 200 reads in at least one sample were considered for testing. *p*-value thresholds were selected such that the significance level was less than 0.001.

### 2.7. Supporting Data Information

The raw genome-wide methylation data have been submitted to the NCBI Sequence Read Archive under accession number PRJNA1088816.

## 3. Results

### 3.1. Summary of Methylome Sequencing

Through WGBS, we obtained the raw data of four hindlimb muscle samples from the hibernation and active periods, with each period’s samples comprising two female Chinese alligators. After quality control, more than 198 million reads were obtained from all samples, with base numbers greater than 4.61 × 10^10^ bp and Q20 greater than 97.93% (Supplementary Table S2), which was sufficient for subsequent analysis. Bisulfite conversion efficiency (BCE) reached 99%, suggesting the reliability of the methylome sequencing in this study.

### 3.2. DNA Methylation Patterns

**Figure 1:**
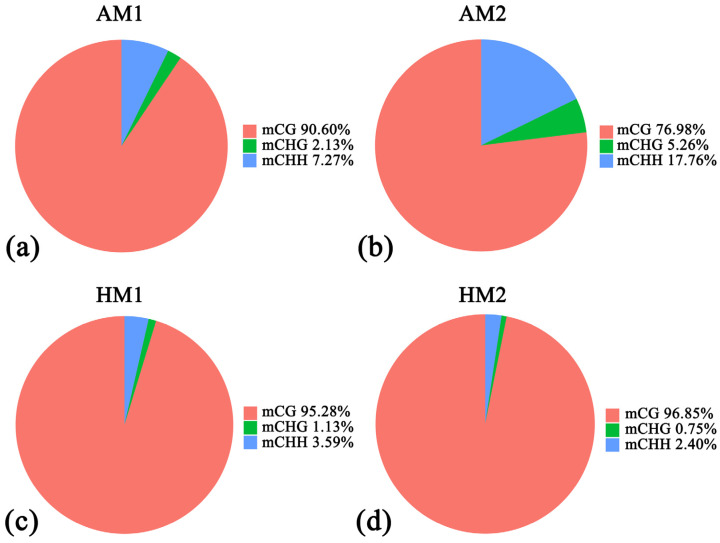
Proportional distribution of methylation C sites. (**a**) AM1: Samples of hindlimb muscles from active period individual 1; (**b**) AM2: Samples of hindlimb muscles from active period individual 2; (**c**) HM1: Samples of hindlimb muscles from hibernation period individual 1; (**d**) HM2: Samples of hindlimb muscles from hibernation period individual 2.

We conducted coverage statistics for C sites, calculating the coverage of C sites in three sequence contexts: CG, CHH, and CHG (i.e., the number of reads supporting that context). The coverage of C sites at 5× was between 0.092909376 and 0.397458967, while at 10×, it ranged from 0.038056356 to 0.127042279. Please refer to Table 1 for specific statistical results. We observed an average methylation rate of 2.97% across all genomic C sites in the four samples. In vertebrates, DNA methylation predominantly occurs within three sequence contexts: CpG, CHG (where H represents A, C, or T), and CHH. The results of this study showed that the methylation ratio of CG sequence types in the hindlimb muscle of a Chinese alligator was 71.12–74.87%. The methylation ratio of CHH sequence type ranges from 0.09–0.84%, and that of CHG sequence type ranges from 0.09–0.83% (Supplementary Table S3). The proportion of methylated C sites (C sites that have at least one methylated read) to all methylated C sites in mCG, mCHG, and mCHH sequences was further analyzed. The results showed that the proportion of CG-methylated C sites was 76.98–96.85%, the proportion of mCHG was 0.75–5.27%, and the proportion of mCHH was 2.40–17.76% (Figure 1), indicating that the methylation of C sites in the mCG sequence context is the predominant type of genomic methylation in the hindlimb muscles of the Chinese alligator.

### 3.3. DNA Methylation Levels in Different Regions of the Gene

**Figure 2:**
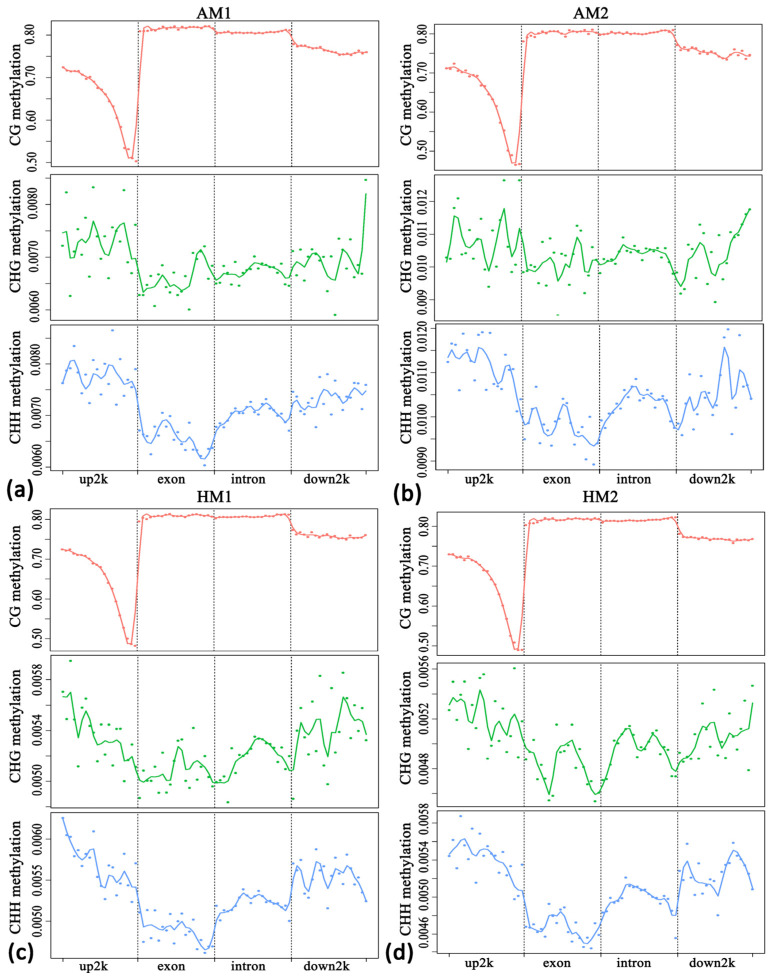
Distribution of sample methylation level in the genebody and upstream and downstream of 2 K. The horizontal axis represents different regions, while the vertical axis represents the level of methylation. Each region of each gene is divided into 20 bins, and then the average level of CpG sites in the corresponding bins of all regions is taken. Different colors represent different sequence contexts (CpG, CHG, and CHH). (**a**) Distribution of sample AM1 methylation level in the genebody and upstream and downstream of 2 K. (**b**) Distribution of sample AM2 methylation level in the genebody and upstream and downstream of 2 K. (**c**) Distribution of sample HM1 methylation level in the genebody and upstream and downstream of 2 K. (**d**) Distribution of sample HM2 methylation level in the genebody and upstream and downstream of 2 K.

To further understand the methylation levels in different regions of the genome, this study targeted three sequence environments (mCG, mCHG, and mCHH) for C sites in the genebody and transcription start sites (TSS) upstream of 2 K. Average methylation levels at 2 K downstream of transcriptional termination sites (TES) were statistically analyzed. As shown in Figure 2, methylation levels of exons and introns were higher in CG sequences than in promoter regions upstream of transcription start sites (TSS) at 2 K, whereas in CHG and CHH sequences, exons and introns have lower methylation levels than the promoter region at 2 K upstream of the transcription start site (TSS). In the context of CHG and CHH sequences, the DNA methylation level in the 2 K region upstream of the transcription start site (TSS) was higher, and the DNA methylation level in the exon region was lower.

### 3.4. Differentially Methylated Regions and Differentially Methylated Gene Identification

**Figure 3:**
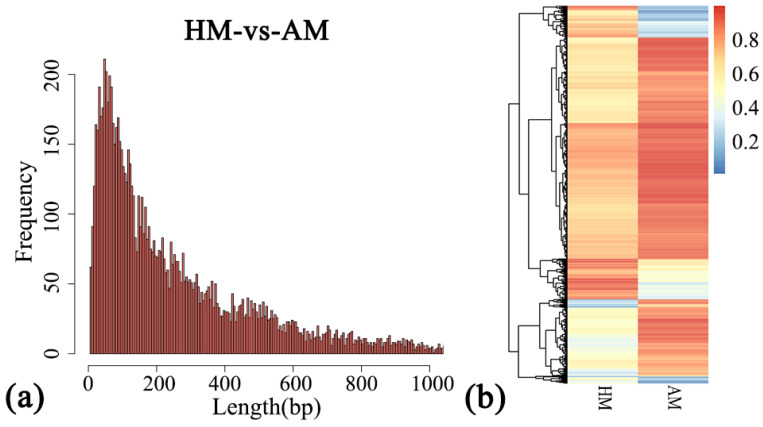
Differential methylation region analysis in the hibernating Chinese alligator. (**a**) Length distribution of DMRs; number of DMRs: 9447. (**b**) Heatmap of methylation levels in the hibernation and active hindlimb muscles comparison group; number of DMRs: 5999; HM includes two samples, HM1 and HM2; AM includes two samples, AM1 and AM2.

In this study, CG sites were used to find differential methylation regions. A total of 9447 differentially methylated regions (DMRs) were detected in the hindlimb muscles of the Chinese alligator during the hibernation and active phase (HM vs. AM) (Supplementary Table S4), corresponding to 2329 genes (Supplementary Table S5). The average length of DMRs is 276, with 88.20% of DMRs being below 600 bp (Figure 3a). According to the average methylation level of the differential methylation region, the cluster heat map of DMRs was plotted (Figure 3b), and there were significant differences in methylation levels at different times. For the DMR anchor region, the DMR number distribution statistics were carried out, and most DMRs were located in the intron region, followed by the promoter region (Supplementary Table S6).

### 3.5. GO Annotation and KEGG Pathway Enrichment on the DMGs and DPMGs

**Figure 4:**
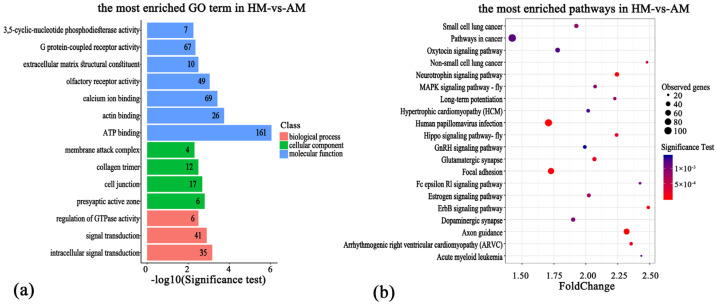
Gene ontology (GO) annotation and Kyoto Encyclopedia of Genes and Genomes (KEGG) pathways enrichment on the differentially methylated genes; number of genes: 2329. (**a**) TOP items by GO enrichment of DMR-related genes. (**b**) TOP items by KEGG enrichment with DMR-related genes.

In order to delve deeper into the potential links between differentially methylated genes (DMGs) and differentially promoter-methylated genes (DPMGs) and the hibernation adaptability of the Chinese alligator, we performed functional enrichment analysis using the clusterprofiler package. Our findings revealed a significant enrichment of DMGs across 79 GO terms (*p* < 0.05; Supplementary Table S7). From these, the most enriched terms included ATP binding, actin binding, calcium ion binding, actin cytoskeleton organization, intracellular signal transduction, olfactory receptor activity, signal transduction, presynaptic active zone, cell junction, regulation of GTPase activity, collagen trimer, extracellular matrix structural constituent, and G protein-coupled receptor activity (Figure 4a).

According to the KEGG analysis, the DMGs were significantly enriched in 36 pathways (*p* < 0.05; Supplementary Table S8). The results showed that pathway enrichment items mainly included the neurotrophin signaling pathway, adhesive patch, ErbB signaling pathway, Hippo signaling pathway, MAPK signaling pathway, oxytocin signaling pathway, GnRH signaling pathway, amino acid biosynthesis, actin cytoskeleton regulation, etc. (Figure 4b).

**Figure 5:**
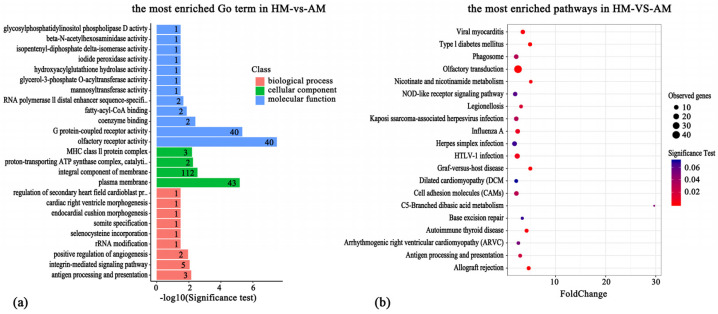
Gene ontology (GO) annotation and Kyoto Encyclopedia of Genes and Genomes (KEGG) pathways enrichment on the differentially promoter-methylated genes; number of genes: 412. (**a**) TOP items by GO enrichment with DMR-related genes of the promoter region. (**b**) KEGG enrichment of TOP entries with DMR-related genes in the promoter region.

To further investigate the potential association of DPMGs with hibernation in the Chinese alligator, 426 DMRs corresponding to 412 genes were screened in the promoter regions of the genome. With regard to DPMGs, 106 GO terms were found to be significantly enriched (*p* < 0.05; Supplementary Table S9), including proton-transporting ATP synthase complex, fatty-acyl-CoA binding, glycerol-3-phosphate O-acyltransferase activity, hydroxyl-glutamine hydrolase activity, pyruvate dehydrogenase (acetyl-transferring) kinase activity, ATP synthesis-coupled proton transport, and other items closely related to energy metabolism. In addition, methyltransferase activity is also one of the significantly enriched GO items (Figure 5a). According to GO enrichment entries and gene functional annotations, genes related to energy metabolism were identified as ATP5F1C, ATP5MD, PDK3, ANGPTL1, and ANGPTL2. Genes related to ubiquitination and proteasome degradation were FBXO28, FBXO43, KLHL40, and PSMD5. These results suggest that promoter methylation may play an important role in the regulation of hibernation adaptability in Chinese alligators.

A total of 92 pathways were enriched (*p* < 0.05; Supplementary Table S10), in which the most significant pathways included graft-versus-host disease, allograft rejection, autoimmune thyroid disease, HTLV-I infection, influenza A, legionellosis, antigen processing and presentation, and phagosome (Figure 5b). These pathways are mostly related to immunity, which may be caused by the difference in disease resistance between hibernating and active Chinese alligators. In some major enrichment pathways, such as thyroid hormone synthesis, apoptosis, glutathione metabolism, and the Notch signaling pathway, there was no significant enrichment. But most of these pathways are related to immune activity, and these pathways may be related to the onset of disease and immune changes during hibernation in alligators.

### 3.6. Bisulfite Sequencing PCR (BSP) Verification Results

**Figure 6:**
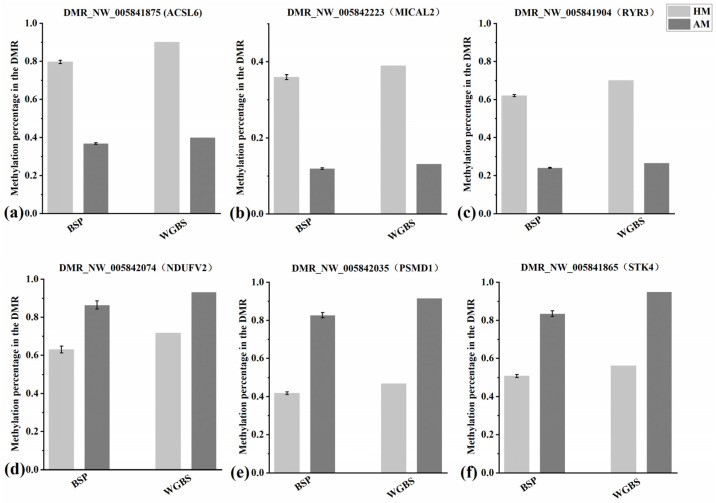
Verification of DMR-associated genes (DMGs) by bisulfite sequencing PCR (BSP). (**a**) Comparison of the degree of methylation of DMR_NW_005841875 (ACSL6) between BSP and whole-genome bisulfite sequencing (WGBS). For BSP, the ordinate represents the mean methylation rate of two samples in each group, while for WGBS, it represents the methylation level of the mean normalized DMR. (**b**) Comparison of the extent of methylation of DMR_NW_005842223 (MICAL2) between BSP and WGBS; (**c**) Comparison of the extent of methylation of DMR_NW_005841904 (RYR3) between BSP and WGBS; (**d**) Comparison of the extent of methylation of DMR_NW_005842074 (NDUFV2) between BSP and WGBS; (**e**) Comparison of the extent of methylation of DMR_NW_005842035 (PMSD1) between BSP and WGBS; (**f**) Comparison of the extent of methylation of DMR_NW_005841865 (STK4) between BSP and WGBS.

Six DMRs (three hypo-methylated DMRs and three hyper-methylated DMRs) were randomly selected from the HM group to verify the reliability of the WGBS data through BSP, namely, DMR_NW_005841875 (ACSL6), DMR_NW_005842223 (MICAL2), DMR_NW_005842074 (NDUFV2), DMR_NW_005842035 (PSMD1), DMR_NW_005841904 (RYR3), and DMR_NW_005841865 (STK4). Compared with the methylation levels observed in the AM group, the HM group exhibited hyper-methylation of ACSL6, MICAL2, and RYR3 (*p* < 0.05). Conversely, the HM group showed hypo-methylation of NDUFV2, PSMD1, and STK4 compared to the AM group (*p* < 0.05) (Figure 6). The methylation analysis of these six DMRs was consistent between BSP and WGBS, indicating that the WGBS data in this study were reliable.

## 4. Discussion

DNA methylation has been reported to play a significant role in regulating gene expression associated with mammalian hibernation [14,15,47]. In thirteen-lined ground squirrels, the global DNA methylation level was increased in brown adipose tissue [15] but decreased in skeletal muscle [14]. The CpG methylation level of the MEF2C promoter region was correlated with the downregulation of gene expression in the skeletal muscle of thirteen-lined ground squirrels [14]. In chipmunks, CpG methylation in the USF-binding site is crucial for liver-specific transcription of the hibernation-specific gene, HP-27 [47]. Although these studies provided a glimpse into DNA regulation in hibernation, they mainly focused on changes in overall DNA methylation levels or the methylation of specific genes.

After analyzing the transcriptome data of hindlimb muscle during hibernation and active periods in Chinese alligators, we observed that the muscle tissue exhibited the highest number of differentially expressed genes [20]. Furthermore, a significant proportion of these differentially expressed genes identified through GO enrichment analysis were found to be associated with the hibernation adaptability of Chinese alligators. Simultaneously, pathway analysis revealed significantly enriched pathways encompassing fatty acid metabolism, starch and sucrose metabolism, ketone body synthesis and degradation, the thyroid hormone signaling pathway, the pentose phosphate pathway, and fatty acid degradation in the muscles of Chinese alligators during hibernation [20]. These findings indicate a multitude of energy regulation processes occurring within their musculature. The differential gene expression in the muscle tissue of Chinese alligators during hibernation plays a critical role in coping with energy deficiency during this period. DNA methylation, as an important epigenetic factor regulating differential gene expression, is speculated to modulate the expression of key genes during physiological changes associated with hibernation. Therefore, in order to understand the association between methylation in the hindlimb muscles of Chinese alligators during hibernation and the physiological changes during this period, genome-wide DNA methylation maps with single-base resolution were generated.

The distribution of methylation sites in functional regions revealed that within the CG sequence context, the genebody region exhibited the highest level of DNA methylation, while the promoter region located at 2 K upstream of the transcriptional start site (TSS) displayed a relatively lower level of DNA methylation. Notably, the promoter proximal region near the TSS exhibited the lowest level of DNA methylation. These findings were consistent with observations in other species [48,49]. This observation suggests that different species exhibit similar DNA methylation patterns and possess certain conserved characteristics. A total of 2329 genes displayed differential methylation in the muscle comparison group during hibernation and active periods, with 412 of these genes exhibiting differential methylation in the promoter region, indicating that the majority of methylation differences occurred within the genebody region. This result is consistent with previous reports indicating widespread methylation within the genebody region [50,51]. The absence of a promoter region may be attributed to the hypomethylation status of CpG sites within the promoter region.

To explore the role of methylation modification in the muscle genome of the Chinese alligator hindlimb, pathway enrichment analysis was conducted on 2329 DMR-related genes. The main pathways of significant enrichment include the neurotrophin signaling pathway, adhesion plaque, ErbB signaling pathway, Hippo signaling pathway, MAPK signaling pathway, oxytocin signaling pathway, amino acid biosynthesis, and regulation of the actin cytoskeleton. The neurotrophin signaling pathway is a cellular signaling mechanism that plays a crucial role in the development, plasticity, and repair of the nervous system [52]. The ErbB signaling pathway regulates cell proliferation, migration, differentiation, apoptosis, and cell motility by mediating the PI3K/Akt signaling pathway, the JAK/STAT signaling pathway, and the MAPK signaling pathway [53]. The Hippo signaling pathway, composed of a set of conserved kinases, is a signaling pathway that inhibits cell growth [54]. The MAPK signaling pathway is primarily involved in the regulation of cell proliferation, growth, and differentiation in response to activation by various growth factors, cytokines, mitogens, and hormone receptors [55]. These pathways may be involved in protecting the brain, reducing metabolism, and controlling growth to conserve energy during hibernation in Chinese alligators.

It has been reported that the methylation modification of the gene promoter region is closely related to gene expression level and complex, and the correlation between them can be positive or negative [56,57,58]. Through GO enrichment entries and gene function annotation, some genes related to the differential methylation region in the promoter region were screened out that were related to energy metabolism and protein degradation. For example, PDK3, pyruvate dehydrogenase kinase 3, through phosphorylation, inhibits pyruvate dehydrogenase activity, thus regulating glucose metabolism and aerobic respiration, causing PDHA2 phosphorylation, reducing glucose utilization, and, at the same time, increasing fat metabolism in response to a long period of fasting, low nutrition level, and starvation state, and playing a role in maintaining glucose homeostasis and normal blood sugar levels [59,60,61]. ATP5F1C and ATP5MD are mitochondrial membrane ATP synthases, which catalyze ADP to produce ATP in the presence of a proton gradient on the cell membrane. ATP5MD is a small subunit of mitochondrial membrane ATP synthase that is required by the dimerization of ATP synthase and that regulates ATP synthesis in mitochondria [62,63]. Both FBXO28 and FBXO43 are members of the F-box protein family that recognize and bind some phosphorylated proteins and promote their ubiquitination and degradation [64]. PSMD5, the 26S subunit of the proteasome, acts as a chaperone protein during the assembly of the 26S proteasome and is directly involved in the ubiquitination proteasome degradation pathway [65,66]. As a key regulatory factor of skeletal muscle development and a gene related to the ubiquitination pathway of protein degradation, KLHL40 is differentially methylated in the promoter region [67], which may play a key role in the synthesis and degradation of hibernating Chinese alligator muscles. In conclusion, it is postulated that differential methylation of the promoter region plays a pivotal role in facilitating hibernation adaptability among Chinese alligators. Significantly enriched pathways in pathway analysis are mainly related to body immunity, which may be associated with the drastic changes in the immune defense capabilities of Chinese alligators during hibernation and active periods. This suggests that the decreased immunity of Chinese alligators during hibernation may exacerbate the survival pressure during this period.

## 5. Conclusions

This study employed whole-genome bisulfite sequencing (WGBS) technology to construct comprehensive DNA methylation maps for the hindlimb muscles of the Chinese alligator during both hibernation and active periods. By comparing the methylation patterns between these two states, 9447 differentially methylated regions (DMRs) and 2329 differentially methylated genes (DMGs) were identified, highlighting extensive epigenetic differences between hibernation and non-hibernation states that are likely related to the alligator’s physiological adjustments to cold environments. The KEGG enrichment analysis of DMGs revealed significant enrichment in several biological pathways, including energy metabolism, amino acid metabolism, and neurotrophin signaling pathways, suggesting that methylation may play a role in energy utilization, substance metabolism, and signal transduction during hibernation in Chinese alligators. This research provides valuable data and insights for understanding the epigenetic mechanisms behind the hibernation of the Chinese alligator.

## Figures and Tables

**Table 1:** Sequencing depth and coverage statistics of the C sites of different samples.

Sample	Context	Total Cytosine	Mean Depth	Effect Depth	Coverage	Coverage (d ≥ 5)	Coverage (d ≥ 10)
HM1	CG	46968104	4.15898675	6.068255263	0.6853678	0.293511954	0.091199125
	CHG	230351287	4.22767468	5.635825908	0.7501429	0.351006951	0.112980272
	CHH	700721371	4.43844499	5.774882225	0.7685776	0.368389289	0.121295292
HM2	CG	46969831	4.20258038	5.700828076	0.7371877	0.302449481	0.088304065
	CHG	230357692	4.41676532	5.493782846	0.803957	0.371469423	0.114224464
	CHH	700738067	4.69871702	5.696217024	0.8248838	0.397458967	0.127042279
AM1	CG	46965911	2.51789803	5.174029373	0.4866416	0.162197918	0.045486097
	CHG	230348012	2.70189185	4.805640649	0.5622334	0.209180338	0.061420725
	CHH	700713976	3.03230192	5.021183223	0.6039019	0.237049672	0.072808576
AM2	CG	46957360	1.6291086	7.736940848	0.2105624	0.092909376	0.038056356
	CHG	230319622	1.53701956	6.548966463	0.2346965	0.114933477	0.048410092
	CHH	700639110	1.73514817	6.767775148	0.2563838	0.129297061	0.055583607

## Data Availability

The datasets used and analyzed during the current study are available from the first author upon reasonable request.

## Supplementary Materials

The following supporting information can be downloaded at: https://www.mdpi.com/article/10.3390/ani14131972/s1, Table S1A: Basic physiological information of the sample; Table S1B: Sample details and numbers; Table S2: Statistics and analysis of filtered data; Table S3: List of whole genome methylations; Table S4: Statistics of DMRs in the HM vs. AM comparison group; Table S5: Differentially methylated regions and differentially methylated genes; Table S6: DMR distribution among different genetic elements; Table S7: GO enrichment of DMR-related genes; Table S8: KEGG enrichment with DMR-related genes; Table S9: GO enrichment with DMR-related genes of the promoter region; Table S10: KEGG enrichment with DMR-related genes of the promoter region. Table S11: Bisulfite sequencing PCR primer information.
